# Rapid 3D Refractive‐Index Imaging of Live Cells in Suspension without Labeling Using Dielectrophoretic Cell Rotation

**DOI:** 10.1002/advs.201600205

**Published:** 2016-10-21

**Authors:** Mor Habaza, Michael Kirschbaum, Christian Guernth‐Marschner, Gili Dardikman, Itay Barnea, Rafi Korenstein, Claus Duschl, Natan T. Shaked

**Affiliations:** ^1^ Department of Biomedical EngineeringFaculty of EngineeringTel Aviv UniversityTel Aviv69978Israel; ^2^ Fraunhofer Institute for Cell Therapy and ImmunologyBranch PotsdamAm Muehlenberg 1314476PotsdamGermany; ^3^ Department of Physiology and PharmacologyFaculty of MedicineTel Aviv UniversityTel Aviv69978Israel

**Keywords:** 3D cell imaging, digital holographic microscopy, microfluidics, quantitative phase microscopy, tomography

## Abstract

A major challenge in the field of optical imaging of live cells is achieving rapid, 3D, and noninvasive imaging of isolated cells without labeling. If successful, many clinical procedures involving analysis and sorting of cells drawn from body fluids, including blood, can be significantly improved. A new label‐free tomographic interferometry approach is presented. This approach provides rapid capturing of the 3D refractive‐index distribution of single cells in suspension. The cells flow in a microfluidic channel, are trapped, and then rapidly rotated by dielectrophoretic forces in a noninvasive and precise manner. Interferometric projections of the rotated cell are acquired and processed into the cellular 3D refractive‐index map. Uniquely, this approach provides full (360°) coverage of the rotation angular range around any axis, and knowledge on the viewing angle. The experimental demonstrations presented include 3D, label‐free imaging of cancer cells and three types of white blood cells. This approach is expected to be useful for label‐free cell sorting, as well as for detection and monitoring of pathological conditions resulting in cellular morphology changes or occurrence of specific cell types in blood or other body fluids.

## 1 Introduction

The ability to identify and characterize different cell types in a heterogeneous medium has an important role in biological and medical research, as well as in clinical practice. Live biological cells are 3D dynamic microscopic objects that constantly adjust their sizes, shapes, and other biophysical features. Visualizing cellular phenomena requires microscopic techniques that can achieve high data acquisition rates, while retaining both resolution and contrast to observe fine cellular features. However, cells in vitro are mostly‐transparent 3D objects with absorbance and reflection characteristics that are very similar to their surroundings, and thus conventional intensity‐based light microscopy approaches lack the required sensitivity. Conventional phase contrast imaging methods, such as Zernike's phase contrast and differential interference contrast, are not quantitative, and present significant imaging artifacts. Indirect cell analyses rely on labeling of a specific cellular entity using exogenous labeling agents, such as fluorescent dyes that tend to photobleach and might damage the sample viability. Still, the widely used methods for detection and diagnosis of diseases such as cancer at the cellular level are based on indirect and subjective histological and cytological examination of tissues or samples from body fluids; procedures which are well known for their inter‐observer variability.

A key effort in the field of biomedical optical imaging is aimed at achieving affordable label‐free but still fully quantitative 3D cellular measurements, which offer high‐resolution and rapid morphological mapping of dynamic cell populations at the single‐cell level. This ability will potentially aid in improving the characterization of the subtle morphological transformations between healthy and diseased conditions for cells drawn from body fluids. This task requires rapid measurements of unattached cells in suspension, typically in flow environment.

To obtain three spatial dimensions in label‐free cell imaging, tomographic interferometry captures the complex wave front of the light transmitted through the cell from various angles, enabling the calculation of the 3D refractive‐index map of the sample. To view the sample from multiple angles, one can rotate the illumination beam, while leaving the measured specimen stationary.^1^, ^2^, ^3^, ^4^, ^5^, ^6^, ^7^ This approach is noninvasive to the sample during data acquisition. However, the acceptance angle of the illumination is limited, typically to 140°, causing missing data points in the angular spectrum. Alternatively, the cells can be imaged during flow, but with limited angular range, and without verified control on the viewing angle.^8^, ^9^ Other approaches allow full angular range, by either rotating the entire sample^10^, ^11^ or patch clamping single cells.^12^ These approaches, however, do not allow for noninvasive 3D imaging of cells in suspension due to unwanted effects on the measured cell or on the surrounding cells. To cope with this barrier, integrating holographic optical tweezers with tomographic interferometry has been proposed lately.^13^, ^14^ This method is able to rotate small and relatively dense cells across 180^o^ range,^13^ but does not allow for a complete rotation of large cells steadily enough. Müller et al.^15^ lately presented preliminary results of diffraction tomography based on the rotation of a cell around a single axis using dual‐beam laser trapping.

In this paper, we present a new approach for interferometric tomography with 360° rotation of single cells in suspension around any axis with angular resolution of less than 2.5°, without physically touching the cells. This allows full coverage of the 3D Fourier spectrum and better tomographic results with isotropic resolution.^16^, ^17^, ^18^ To obtain this challenging goal, we integrated interferometric imaging of cells flowing in a microfluidic channel, while being trapped, manipulated, and fully rotated by dielectrophoretic forces (DEP).^19^, ^20^, ^21^ DEP can be utilized for cell micromanipulation and detection of inherent cellular traits such as membrane capacitance, electrical conductivity, nucleic acid content, cell size, and deformability, which can then be utilized for cell sorting.^22^, ^23^, ^24^, ^25^, ^26^ Microfluidics combined with DEP has the superior ability to manipulate small volumes of liquids while trapping and controlling live cells noninvasively.^25^, ^26^, ^27^ The combination of DEP and tomographic interferometry provides an elegant way to fully capture the 3D refractive‐index map of single cells, temporally trapped in a flowing environment, in a noninvasive manner, and with control on the viewing angle.

## 2 Projection Mapping in the 3D Fourier Space

**Figure 1:**
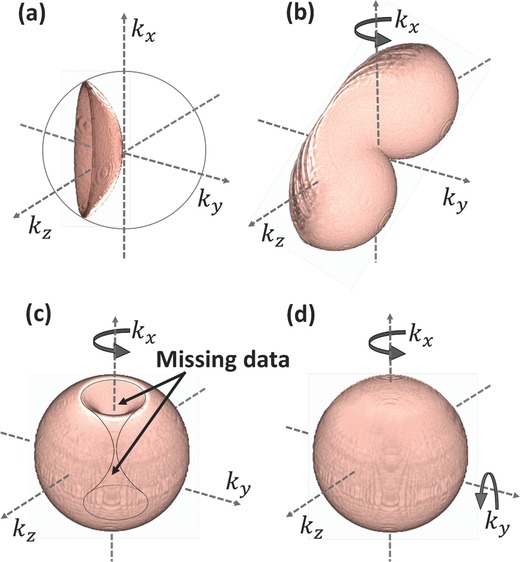
Mapping of the angular projections in the 3D Fourier space using the diffraction‐theory method in the case of: a) a single projection, b) angular range of 140° around one axis by illumination rotation, c) angular range of 360° around one axis by sample rotation, d) angular range of 360° around two axes by sample rotation, presenting the most complete angular range possible. To create these schematic images, 3D iso‐surfaces of half hemispheres in multiple angles were simulated using Matlab, saved in nii.gz format, and the final renderings were created by MRIcroGL64 software.

**Figure** **1** demonstrates the 3D Fourier‐spectrum coverage of mapping the interferometric projections, comparing the previous methods and the proposed one, and signifying the advantage of full cell rotation around two axes. Figure 1a shows the mapping of the angular projections in the 3D Fourier space using the diffraction‐theory method in the case of a single projection. Figure 1b shows the mapping for an angular range of 140° when using the illumination‐rotation method, where 140° is the typical acceptance‐angle limit. In this case, the mapping is smeared and there are many missing data points in the 3D Fourier space.

Figure 1c shows the mapping for an angular range of 360° when using the sample‐rotation method. This was implemented so far by physically mounting the entire sample to a rotation stage or patch clamping a single cell and mechanically rotating it, which is slow, cell‐invasive and cannot be implemented for many cells in suspension. In addition, there are missing data points composing togther a shape of two parabolic cones with a common edge.

Figure 1d shows the mapping for an angular range of 360° around two axes, presenting a complete coverage of the angular range in the 3D Fourier space. For the first time, this complete tomographic mapping can be implemented rapidly by the DEP/interferometry method presented in this paper for cells in suspension in a noninvasive manner.

**Figure 2:**
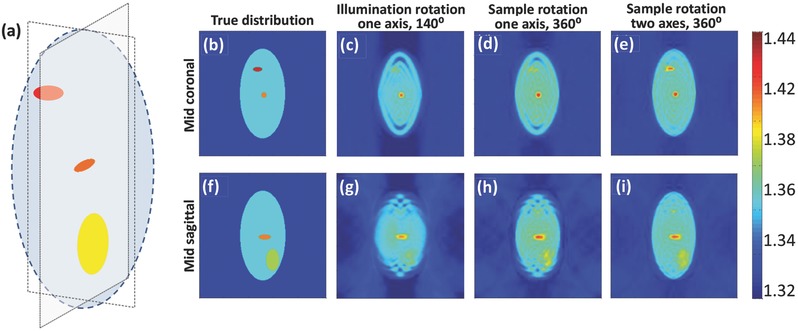
A comparison between the 3D refractive‐index reconstructions obtained by tomographic simulations. a) The input 3D refractive‐index model used. b–e) Mid coronal section of the refractive‐index distribution for: b) the true (input) values, c) the reconstructed values for the illumination‐rotation method, around one axis with 140° angular range, d) the reconstructed values for the sample‐rotation method, around one axis with 360° angular range, e) the reconstructed values for the sample rotation method, around two axes with 360° angular range. f–i) Mid sagittal section of the refractive‐index distribution for the coinciding cases to (b)–(e), respectively. Color bar represents refractive‐index values.

To verify the advantage of being able to fully rotate a cell around two orthogonal axes and cover the entire 3D Fourier domain, we conducted a tomographic simulation based on a known refractive‐index distribution of 3D test target shown in Figure 1a. The simulation, implemented in Matlab, imitated an interferometric system with straight light propagation, where the phase delay was calculated as the integration over the refractive index of the sample, and the amplitude was assumed to be unit (a pure‐phase sample). The angular increment was assumed to be 1°. All reconstructions were based on the Rytov approximation for optical diffraction tomography.^3^ The reconstruction qualities in various cell rotation scenarios are compared in **Figure** **2**.

In the first scenario, we imitated acquisition by illumination rotation around one axis,^3^ with limited angular coverage of 140°, the angular spectrum coverage of which is shown in Figure 1b and the coinciding reconstructed refractive‐index profile is shown in Figure 2c,g. In this case, the total phase delay was composed of both the phase delay induced by the sample and the phase ramp introduced by the tilted illumination, and captured from multiple angles. In the second scenario, we imitated acquisition by full rotation of the cell around one axis (360° range), the angular spectrum coverage of which is shown in Figure 1c and the coinciding reconstructed refractive‐index profile is shown in Figure 2d,h, as can be provided by the proposed method when rotating around one axis. In the third scenario, we imitated acquisition by full rotation of the cell around two axes (360° range, twice), the angular spectrum coverage of which is shown in Figure 1d and the coinciding reconstructed refractive‐index profile is shown in Figure 2e,i, as can be provided by the proposed method when rotating the cell around two axes. As shown in Figure 2e,i, all organelles present in the input distribution (Figure 2b,f, respectively) are distinctly visible when performing reconstruction based on sample rotation around two axes. In the reconstruction based on cell rotation around one axis only (Figure 2d,h), the upper organelle in the mid coronal section is hardly seen, and the lower organelle in the mid sagittal section is blurry. This is a direct result of the missing data points indicated in Figure 1c. Finally, in the reconstruction based on illumination rotation with 140° angular coverage (Figure 2c,g), due to the missing angles only one of the two organelles is visible and the second one, as well as the edges of the large shape, are heavily blurred. This signifies the advantages of being able to fully cover the 3D spatial Fourier domain by using cell rotation around two axes. This is obtained here, for the first time for our knowledge, by combining tomographic interferometry with microfluidics and DEP to allow full angular coverage for suspended cells.

## 3 Hybridizing Tomographic Interferometry with Microfluidics and DEP

**Figure 3:**
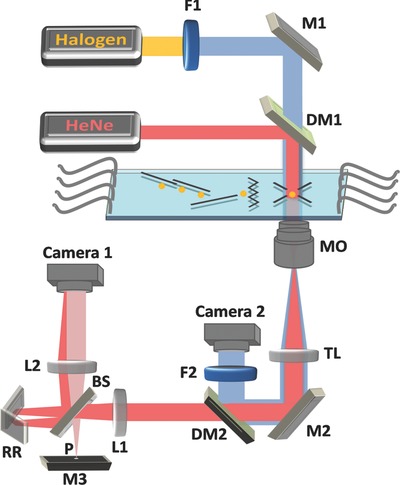
Optical setup scheme for interferometric tomography with full rotation of single trapped cells in a microfluidic flow environment. The system combines a microfluidic DEP‐based device for cell rotation and an external interferometric module for acquisition of off‐axis interferograms during cell rotation. From these interferometric projections, the cell complex wave front can be reconstructed. Bright‐field microscopy is used as a control imaging system. Red beams: Interferometric microscopy; Blue beams: Bright‐field microscopy; HeNe: Helium‐neon laser; Halogen: tungsten‐halogen lamp; MO: Microscope objective; TL: Tube lens; F1, F2: Bandpass filters; M1–M3: Mirrors; DM1‐DM2: Dichroic mirrors; L1, L2: Lenses in 4f configuration; BS: Beam splitter; RR: Retro‐reflector; P: Pinhole.

**Figure** **3** shows a scheme of the proposed interferometry/DEP integrated system. This system includes two imaging channels: interferometric microscopy for acquisition of off‐axis image interferograms during cell rotation, and bright‐field microscopy for imaging the entire microfluidic channel and controlling the cell trapping and rotation. In the interferometric microscopy channel, light from a helium‐neon laser is reflected to the sample by dichroic mirror DM1, and then magnified by a 60× immersion‐oil microscope objective. The enlarged image is projected by tube lens TL onto the exit of the microscope, where an external off‐axis interferometric module is positioned.^28^, ^29^ In this module, the magnified sample beam is split using beam splitter BS. One of the beams is spatially filtered using lenses L1 and L2 and pinhole P that selects only the low spatial frequencies and, thus, effectively creates a reference beam that does not contain spatial modulation from the sample. The other beam from BS is projected through retro‐reflector RR at a small angle, and together with the reference beam creates an off‐axis interferogram on Camera 1. From this interferogram, the complex wave front of the cell at the present viewpoint can be reconstructed (see the Experimental Section for details). In the bright‐field imaging channel a tungsten‐halogen lamp is spectrally filtered using bandpass filter F1, projected onto the sample, magnified by either a 10× or a 60× immersion‐oil microscope objective, and projected through tube lens TL, dichroic mirror DM2, and spectral bandpass filter F2 onto Camera 2.

**Figure 4:**
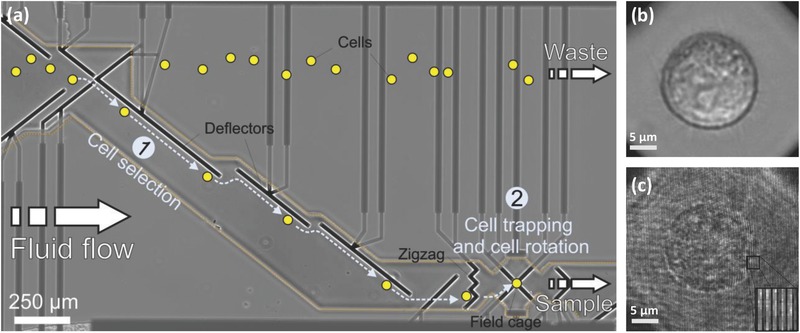
Hybrid imaging in the DEP‐based microfluidic system. a) Microelectrode design and cell handling procedure in the microfluidic channel. Cells are represented by yellow spots. Electrodes from various DEP elements in the channel are marked in black. Electrode supplies are marked in dark gray and are passivated by a Si_3_N_4_ passivation layer (border line of passivation layer is marked by an orange dotted line). The DEP elements include deflectors and zigzag electrodes for laterally moving and retaining cells in the fluid flow, respectively. These elements allow the selection of a single cell from the bulk solution [Area (1)] to be trapped in the eight‐electrode field cage [Area (2)]. There, it is rotated around the axis of choice. The analyzed cells can be released from the channel via the sample outlet, whereas unprocessed cells are discarded to the waste outlet. b) Bright‐field microscopy image of an MCF‐7 cancer cell trapped in the field cage. Supplementary Video 1 (Supporting Information) shows bright‐field imaging of the cell handling in the channel and the rotation of the selected cell in the DEP field cage. c) Off‐axis image interferogram of an MCF‐7 cancer cell trapped in the field cage. The enlarged image at the bottom‐right shows the high‐spatial‐frequency off‐axis fringes from a selected area. Supplementary Video 2 (Supporting Information) shows the cell off‐axis interferogram from multiple directions of view during cell rotation in the DEP field cage.

We recorded suspended cells flowing in a 35 μm high microfluidic channel. The channel comprised several fluid ports that were used to either inject suspended cells/medium, or served as waste outlets. As shown in **Figure** **4**a, the microchannel was equipped with several DEP electrodes (shown in black). These include deflector elements for moving the cells diagonally to the fluid flow, a zigzag element for retaining a chosen cell against the fluid flow, and a DEP field cage for rotating the cell. The DEP field cage comprised eight electrodes, four of which were located below the cell (on the bottom of the microchannel) and four of which were located above the cell (on the top of the microchannel). Selecting the phase shifts between the external radio‐frequency signals applied to these eight electrodes controlled the direction of rotation of the trapped cell around any axis of choice, with a measured axis error of 0.47% (see the Experimental Section). The cycle time for a full cellular rotation is a function of the applied voltage, the frequency of the field, as well as the size and the dielectric function of the cell. It was set to 10–14 s in our experiments. Slight variations of cell parameters were taken into account by a readjustment of voltage and frequency according to prior optical bright‐field inspection of the cells in the cage. It should be noted that cycle times of less than a second are easily achievable without harming the cells. As the DEP force that acts on a cell scales with its volume, the voltage and the frequency were chosen such that the system tolerated naturally occurring variation of the cell morphology within a given population.

Concerning potential effects of DEP on the measurements, the frequency‐dependent complex dielectric constants of the materials of the cell and the aqueous environment determine the response of the cell to external electromagnetic fields. However, the frequencies of the optical field and the field used for DEP manipulation in the MHz range are far apart such that interference can be excluded. In addition, the intensity of the DEP field used is far below the threshold for electroporation leading to protein leakage to the extracellular milieu or bringing about an osmotic volume change due to increased permeability for ions. For these reasons, we do not expect that the DEP field will affect the measured refractive‐index distribution. Given the density differences between the cellular organelles and cytosol and the rotation velocity, centrifugal or shear forces on the cells are negligible as well.

Figure 4b shows a bright‐field image of an MCF‐7 cancer cell in the DEP cage, and Supplementary Video 1 (Supporting Information) shows cell trapping, which is followed by full rotation around two axes. Figure 4c and Supplementary Video 2 (Supporting Information) show the off‐axis interferogram of the cell, acquired by the external interferometric module shown in Figure 3.

## 4 Results and Discussion

### 4.1 Accuracy and Resolution Evaluation

**Figure 5:**
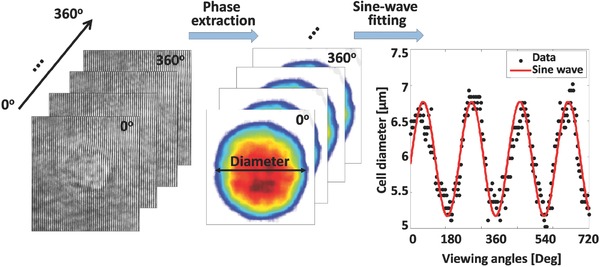
Detection of the rotation cycle time and evaluation of the angle of the present point of view are done by fitting the cell diameter in the quantitative phase map during cell rotation to a sine wave.

For each cell rotating in the DEP cage, a video of off‐axis interferograms was recorded using the external interferometric module. Following the acquisition, the phase map from each interferometric projection was digitally extracted by digital spatial filtering (see the Experimental Section). Based on binary image thresholding of the phase map, both the center of mass of the cell and its diameter were traced. Since cell shapes are not perfect spheres, the diameter detection presented a sinusoidal pattern over the rotation time, as seen in **Figure** **5**. By fitting the cell diameter values from the cell projections to a sine wave, with coefficient of determination of *R* ^2^ = 0.85, the frequency of cell rotation was determined. For the experimental demonstrations, 150 interferometric projections were taken from a complete angular range of 360° and in equally discrete increments. All phase projections were then processed digitally to create the 3D refractive‐index map of the cell by both the filtered back projection and the diffraction‐theory reconstruction algorithms.^13^, ^30^ In this reconstruction process, each projection is mapped to a surface in the 3D Fourier space, where the full rotation provided by DEP enables a full angular coverage of the Fourier space, in contrast to previous methods possessing limited angular range^1^, ^2^, ^3^, ^4^, ^5^, ^6^, ^7^ (see comparison in Figure 1).

For the 3D refractive‐index profile acquired by the proposed method, we performed an evaluation of the refractive‐index resolution, as done in Choi et al.,^1^ by spatial derivations of the 3D refractive‐index map and determining the full‐width at half‐maximum. This resulted in (*y*,*z*) and *x* spatial refractive‐index resolutions of 0.31 and 0.4 μm, respectively (when rotating the cell around *x*).

### 4.2 Label‐Free 3D Refractive‐Index Imaging of Suspended Cells

**Figure 6:**
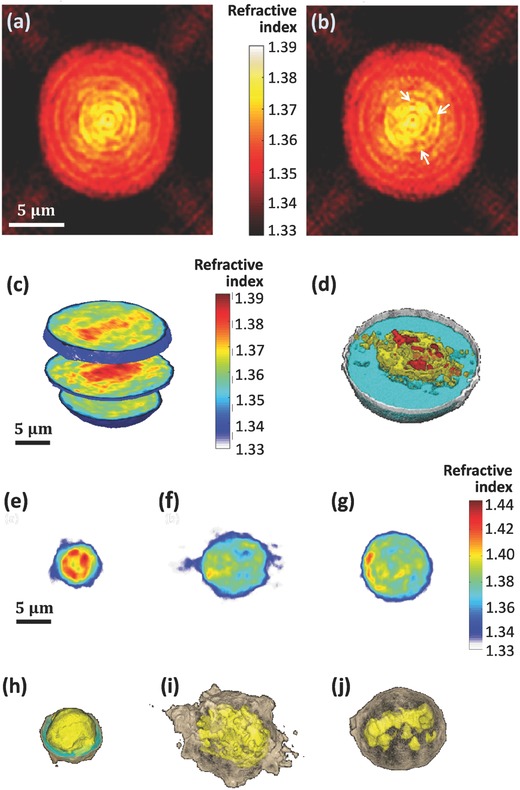
a,b) Refractive‐index maps of an MCF‐7 cell at the mid‐sagittal slice for using a full cell rotation around a single axis (a) and around two axes (b). The white arrows indicate details that are clearer when rotating the cell around two axes. c,d) 3D rendering (c) and rendered iso‐surface plot (d) of the refractive‐index map of an MCF‐7 cancer cell. e–j) Refractive‐index maps of three types of white blood cells at the mid‐axial positions (e–g), and the coinciding rendered iso‐surface plots of the refractive‐index maps (h–j); (e,h) T cell, see also Supplementary Video 4 (Supporting Information); (f,i) Monocyte, see also Supplementary Video 5 (Supporting Information); (g,j) Neutrophil, see also Supplementary Video 6 (Supporting Information).

**Figure** **6**a–d shows the reconstructed refractive‐index maps of large MCF‐7 human cancer cells in suspension, as acquired by interferometry during DEP rotation in the microfluidic channel. First, Figure 6a,b compares the refractive‐index maps of an MCF‐7 cell at the mid‐sagittal slice when using a full cell rotation around a single axis (a) and around two axes (b). The white arrows in Figure 6b indicate spatial details that are clearer when rotating the cell around two axes. Figure 6c,d shows 3D refractive‐index map renderings of the MCF‐7 cell. In this figure, it is possible to distinctively see the cell shape, cytoplasm, and nucleosomic zones.

**Figure 7:**
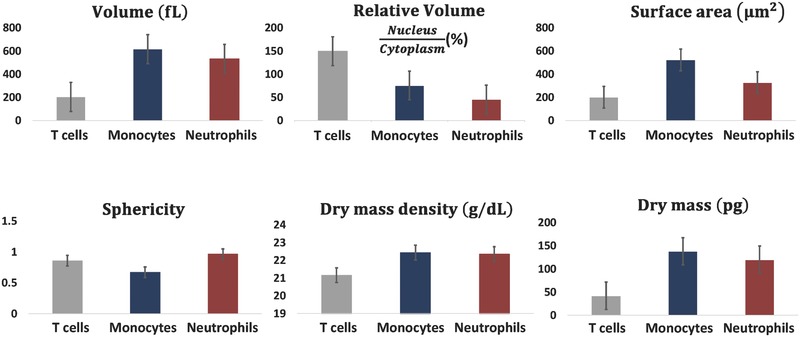
Parameter sets of three types of live white blood cells in suspension, which are based on the calculation of the cell 3D refractive‐index maps obtained by the proposed interferometry/DEP approach.

The proposed technique was also used to acquire three types of white blood cells, illustrating its potential for label‐free cell sorting following a blood test. The resulting 3D refractive‐index map of a T‐cell is shown in Figure 6e,h. As can be seen in these figures, these cells are relatively small and have a large volume of the nuclear zone,^31^, ^32^ in comparison to the monocyte shown in Figure 6f,i. As can be seen in the latter figure, monocytes present a relatively large and a less spherical shape, with larger nuclear and cytoplasmic volumes. In contrast, neutrophils, shown in Figure 6g,j, are spherical and present a large cytoplasmic volume, corresponding to previous label‐based imaging studies.^6^ **Figure** **7** presents various parameters for the three types of white blood cells measured. These include the cell total volume, nucleus to cytoplasm volume ratio, surface area, sphericity, dry mass density, and total dry mass. Note that different types of white blood cells have large variability in the values of these parameters. The nucleus and cytoplasm volumes were discriminated in 3D based on the characteristic cell refractive‐index values (T cells: nucleus, 1.4045 ± 0.0087, cytoplasm, 1.3748 ± 0.0089; monocyte: nucleus, 1.3949 ± 0.0050, cytoplasm, 1.3777 ± 0.0085; neutrophils: nucleus, 1.4061 ± 0.0108, cytoplasm, 1.3759 ± 0.0091). The nucleus refractive‐index values presented above for white blood cells are the averaged refractive‐index values on the entire nucleus, including the nucleoli. These values are higher than these obtained in previous reports for other types of larger cells,^1^, ^11^ in which the nucleoli area is excluded. Another possible reason is that white blood cells are small cells, in which the DNA of the nucleus is more condensed, and thus the refractive index of the nucleus in these cells is expected to be higher in comparison to other types of larger cells. In any case, our morphological results for the white blood cells coincide with those obtained by another group, which used the illumination rotation method,^6^ and the nucleus morphology we obtained for each of the white blood cell types coincides with previous DNA staining results in white blood cells.^31^, ^32^

We have focused here on the new 3D, label‐free cell imaging modality for cells trapped in flowing conditions, rather than cell sorting. In the future, however, this technique might be integrated with sorting of cells of specific types based on decisions made on the resulting 3D cellular analysis. Still, sorting of a very large number of cells, such as separation of white blood cells from the other blood components in high throughput is out of the scope of the current technique.

## 5 Conclusion

In summary, we have developed a new label‐free tomographic imaging approach for 3D refractive‐index mapping of unattached live cells in suspension, while temporally trapped and rotated. To our knowledge, this is the first time that tomography for 3D label‐free imaging of cells in suspension with full angular coverage is experimentally obtained. The proposed system integrates wide‐field interferometry for quantitative phase map acquisition with a microfluidic channel for cell flowing, trapping, and full rotation based on DEP. In contrast to previous approaches for 3D refractive‐index capturing using tomography, our approach is the first one to allow full experimentally measured tomographic interferometry for cells in suspension, with complete rotation around any axis, and with knowledge of the angle of the projection at a given time. It should be emphasized that during recording of data, cells experience no physical contact and only very moderate light exposure. Both features minimize adverse phenomena that could affect the status of the cells or their viability. The suspended cell rotation was steady enough, with less than 0.5% error, to allow tomography with full rotation of both large cells, such as cancer cells, and small cells, such as white blood cells, as experimentally demonstrated. The proposed tomographic acquisition is fast and noninvasive, in contrast to previous interferometric tomography approaches for cells in suspension. These results illustrate the potential of our method as an integrated rapid approach for 3D cell label‐free imaging and analysis. Note that our method is not limited to a single cell rotation at a time, since multiple field cages can be used. Hence, the integrated DEP system can be used for cell sorting, following decisions based on the label‐free 3D imaging.

With the availability of fast acquisition, fast tomographic processing is required for real‐time visualization and decision. Regarding the computational burden, our group has recently presented efficient interferometric phase extraction algorithms, including phase unwrapping, reaching beyond video rate, even on the main processing unit of a conventional personal computer,^33^, ^34^ as well as full processing in tomographic interferometry in video‐rate using the graphics processing unit (GPU) of the computer.^35^

Due to its noninvasiveness and the straightforward recovery of cells after inspection, the new experimental approach presented in this paper is expected to pave the way for label‐free cell sorting, monitoring cellular pathological conditions in body fluids and especially in blood, as well as for therapeutic purposes.

## 6 Experimental Section

*Cell Preparations*: Human breast adenocarcinoma cells from MCF‐7 cell line, purchased from the Deutsche Sammlung von Mikroorganismen und Zellkulturen (DSMZ), were seeded at cell densities of 8 × 10^3^ cm^−2^, cultivated in standard glucose Dulbecco's Modified Eagle Medium (DMEM, Gibco Invitrogen, Carlsbad, CA, USA), supplemented with 10% fetal bovine serum (Biochrom, Berlin, Germany) and incubated at 37 °C under 5% CO_2_. After 4 d of cultivation, cells were harvested by incubation in trypsin/ethylenediaminetetraacetic acid (EDTA) (0.25%/0.02%) for 15 min, and then washed and resuspended in phosphate‐buffered saline (PBS) with Ca^2+^/Mg^2+^.

Human white blood cells were separated from fresh blood obtained from a donor. 10 mL of EDTA‐supplemented blood were added within 2 mL of HetaSep (StemCell Technologies, Columbia, Canada), and centrifuged at 85 g for 7 min. The upper phase of the blood was transferred to a fresh tube diluted with 40 mL of washing buffer, and centrifuged with 350 g for 7 min. After removing the supernatant, the pellet was suspended in 10 mL of daily buffer and centrifuged at 133 g continuously for 7 min. Once more after removing the supernatant, 1 mL of buffer was added and the cells were maintained in 4 °C at a concentration of 5 × 10^7^ cells per mL in tubes for the isolation of the different leucocyte sub‐populations. Monocytes, neutrophils, and T‐cells were isolated by negative selection with magnetic beads using human monocytes, neutrophils, and T‐cell enrichment kits (EasySep, StemCell Technologies, Columbia, Canada), respectively, in accordance with manufacture instructions.

*DEP‐Based Microfluidic System and Cell Handling*: The fabrication process of the microfluidic systems is fully described by Kirschbaum et al.^21^ Briefly, a 35 μm thick SU‐8 polymer spacer was sandwiched between two glass slides of 170 and 700 μm thickness, respectively, so that the resist formed the sidewalls of a microchannel (GeSiM mbH, Grosserkmannsdorf, Germany). The inlets and outlets of the fluidic system were connected with syringe pumps (neMESYS, Cetoni GmbH, Germany) via standard HPLC tubing that allowed controlling the fluid flow in the microchannels. Fluids and cells were introduced into the channel system through separate inlets and transported under laminar flow conditions. The inner faces of the top and bottom slides of the channel were equipped with congruent, 15 μm wide and 120 nm thick platinum microelectrodes. A multichannel radio‐frequency generator (Cytocon 400, Evotec Technologies GmbH, Hamburg, Germany) was used to apply phase‐shifted AC voltage signals of 2–2.5 V at frequencies between 0.9 and 2.25 MHz to each of the electrodes of the DEP elements. The geometry of the electrodes and the signal supply determine the distribution of the electric field and the corresponding potential.

**Figure 8:**
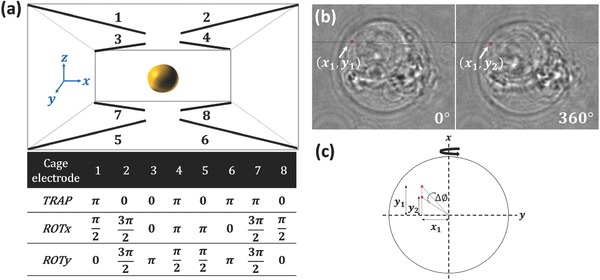
a) Phase shifts applied to the eight electrodes of the DEP field cage for cell trapping (*TRAP*), (negative) rotation around *x* axis (*ROTx*), and (negative) rotation around *y* axis (*ROTy*). b) Bright‐field images of an MCF‐7 cancer cell, where the white arrow indicates a small object traced during the full cell rotation. c) Scheme of the trigonometric calculation of the rotation error around the *y* axis.

As seen in Figure 4 and Supplementary Video 1 (Supporting Information), cells were directed to the zigzag electrode by the deflector electrodes, and were subsequently forwarded to the DEP field cage. For trapping or rotating the cells around a selected axis, the eight electrodes of the DEP field cage were driven at different patterns of 180° or 90° phase‐shifted radio‐frequency signals. The signal patterns used are summarized in **Figure** **8**a. After data acquisition, the cage electrodes were turned off and the cell was flushed out in order to record a background image. We used PBS as the medium in the channel.

The exposure of cells to electric fields may affect the cell physiology due to ohmic warming, toxic compounds electrochemically generated at the electrodes or electrically induced shifts of the membrane potential. We have extensively discussed these issues for a similar experimental setup previously.^36^ Based on these studies, we are confident for a number of reasons that DEP effects on cells are negligible in our experiments: (i) We employ negative DEP for cell guiding and cell rotation.^37^ In this scheme, cells experience forces along the field gradient toward low electric field strength. As a consequence, the cells, when positioned in the centre of our DEP field cages, experience minimal field strength. (ii) We apply radio‐frequency signals in the MHz range to the electrodes, which minimizes possible effects on the membrane potential or electrochemistry at the electrodes. (iii) As shown by thermometry and numerical simulations, ohmic warming in our system is sufficiently low in order to keep the temperature in the microchannel within the physiological range.^36^, ^38^ (iv) Previous experimental data from our group and others show that negative DEP only minimally affects cell viability. It has been used in context of live cell sorting,^25^ immune cell activation,^24^ and in particular for sorting embryonic stem cells, which later contributed to normal mice development.^39^

*Evaluation of DEP Rotation Accuracy*: We applied an external field in order to rotate the cell around the primary axis of choice *x*. However, a random movement of the cell around a secondary axis (*y*) creates imperfect rotation around the desired axis. In order to determine the accuracy of rotation around a single axis, we traced a small object that was visible in the inner portion of the cell over several full rotations, as illustrated in Figure 8b. The average rotation deviation around the y axis was measured to be 3 pixels, which can be translated to degrees according to the following trigonometrical calculation (see Figure 8c)

(1) $$\Delta \phi =\tan^{-1}\left(\frac{y_{1}}{x_{1}}\right)-\tan^{-1}\left(\frac{y_{2}}{x_{1}}\right)=1.69^{{}^{\circ}}$$

We therefore conclude that the relative rotation error between the secondary axis and the main axis is 1.69°/360° = 0.47%. This behavior was repetitive over several cell rotations.

*Interferometric Phase Microscopy*: Interferometric projections from various points of view of a trapped cell, as obtained during DEP rotation, were acquired for tomography. We used a helium‐neon laser (632.8 nm, 5 mW, Thorlabs) as the light source of an inverted microscope (Olympus IX81), for imaging the trapped cell in the DEP microfluidic channel in transmission mode. As shown in Figure 3, the beam was reflected by dichroic mirror DM1 (short‐pass, cut off at 550 nm, Edmunds Optics) onto the sample plane and propagated through a microscope objective (Olympus PlanApo, 60×, 1.4 NA, immersion oil). At the exit of the imaging system, we integrated an off‐axis interferometer. In this external interferometric module, the image plane is optically Fourier transformed by lens L1 (f = 7.5 cm), and split into two separate beams by beam splitter BS. One of the beams (referred to as the reference beam) propagates towards pinhole P (diameter of 15 μm), which spatially filters the beam and thus erases the high spatial frequencies of the sample, turning it into a reference beam. Mirror M3 reflects the filtered beam, and then lens L2 (f = 7.5 cm) Fourier transforms it back onto the camera plane. The other beam at the exit of beam splitter BS (referred to as the sample beam) is reflected back using retro‐reflector RR, which shifts the center of the spatial‐frequency domain. Due to this shift, the sample and reference beams interfere at a small angle on Camera 1 in Figure 3 (CMOS camera, DCC1545, Thorlabs, with 1024 × 1280 square pixels, 5.2 μm each). The off‐axis angle between the beams was set so that a fringe interference cycle will contain 3 pixels. The resultant off‐axis interferogram allows reconstruction of the complex wave front of the sample from a single exposure. In the digital reconstruction process, a discrete Fourier transform is applied on each of the acquired off‐axis interferograms using Matlab. Then, one of the cross‐correlation terms, shifted from the center of the spatial‐frequency domain, is digitally cropped and an inverse Fourier transform is applied to the result. This yields a complex matrix, for which the phase argument is the wrapped phase map of the sample. The same process is performed on a sample‐free interferogram, and the resultant wrapped phase map is subtracted from the wrapped phase map of the sample. Applying a 2D phase unwrapping algorithm on the result solves 2π ambiguities, and yields the unwrapped phase map of the sample from a single point of view of the cell. Then, tomographic mapping into the 3D Fourier space and calculation of the 3D refractive‐index map of the cell are performed, as explained above.

*3D Refractive‐Index Map Based Parameters*: Following the reconstruction of the 3D refractive‐index map, we calculated various quantitative parameters for white blood cells. The cellular volume *V* was obtained by counting the number of voxels inside the 3D refractive‐index map of the cell multiplied by the voxel size. Cytoplasm and nucleus volumes were calculated in the same way as the total volume, but at the locations of low and high refractive indices, respectively (the values of which are elaborated for each cell type in Section 4). The surface area *S* was calculated after locating the boundary of the cellular 3D refractive‐index map. The sphericity of the cell is defined as follows: π^1/3^(6 V)^2/3^/*S*. The dry mass density was calculated as follows: ρ = (*n* – *n*_m_)/α where *n* is the mean refractive‐index value of the cytoplasm, *n*_m_ is the refractive index of the surrounding medium and α is the specific refractive index increment and set to 0.2 mL g^−1^.^6^ Integration of the total dry mass density over the cellular volume yields the cell dry mass.

*Bright‐Field Microscopy*: Bright‐field microscopy was used as a control system, in order to view the trapped cells, carry out a rough evaluation of the rotation cycle time, and validate that the rotation is performed around the axis of choice. In this imaging channel, light from a tungsten‐halogen lamp was filtered through a band pass filter F1 (475/35 nm), transmitted through the sample and through one of the following microscope objectives: Olympus PlanApo, 10×, 0.25 NA objective for imaging the microchannel in a wide field of view, or Olympus PlanApo, 60×, 1.4 NA, oil‐immersion objective for imaging only the cell in the DEP field cage. Dichroic mirror DM2 (long‐pass, 505 nm, Edmunds Optics) reflected the bright‐field image through bandpass filter F2 (475/28 nm) onto Camera 2 (Olympus F‐View II, 1376 × 1032 pixels, 6.45 μm each), as can be seen in Figure 3.

## Supporting information

As a service to our authors and readers, this journal provides supporting information supplied by the authors. Such materials are peer reviewed and may be re‐organized for online delivery, but are not copy‐edited or typeset. Technical support issues arising from supporting information (other than missing files) should be addressed to the authors.

Supplementary

Supplementary

Supplementary

Supplementary

Supplementary

Supplementary

Supplementary

## References

[1] W. Choi , C. Fang‐Yen , K. Badizadegan , S. Oh , N. Lue , R. R. Dasari , M. S Feld , Nat. Methods 2007, 4, 717. 17694065 10.1038/nmeth1078

[2] S. O. Isikman , W. Bisharaa , S. Mavandadia , F. W. Yua , S. Fenga , R. Laua , A. Ozcan , Proc. Natl. Acad. Sci. USA 2011, 108, 7296.21504943

[3] K. Kim , H. Yoon , M. Diez‐Silva , M. Dao , R. R. Dasari , Y. Park , J. Biomed. Opt. 2014, 19, 011005. 23797986 10.1117/1.JBO.19.1.011005 PMC4019420

[4] Y. Sung , W. Choi , C. Fang‐Yen , K. Badizadegan , R. R. Dasari , M. S. Feld , Opt. Express 2009, 17, 266. 19129896 10.1364/oe.17.000266 PMC2832333

[5] W. C. Hsu , J. W. Su , T. Y. Tseng , K. B. Sung , W. C. Hsu , J. W. Su , T. Y. Tseng , K. B. Sung , Opt. Lett. 2014, 39, 2210. 24686713 10.1364/OL.39.002210

[6] J. Yoon , K. Kim , H. Park , C. Choi , S. Jang , Y. Park , Biomed. Opt. Express 2015, 6, 3865. 26504637 10.1364/BOE.6.003865 PMC4605046

[7] Y. Cotte , F. Toy , P. Jourdain , N. Pavillon , D. Boss , P. Magistretti , P. Marquet , C. Depeursinge , Nat. Photonics 2013, 7, 113.

[8] N. Lue , W. Choi , G. Popescu , K. Badizadegan , R. R. Dasari , M. S. Feld , Opt. Express 2008, 16, 16240. 18825263 10.1364/oe.16.016240 PMC2750801

[9] Y. Sung , N. Lue , B. Hamza , J. Martel , D. Irimia , R. R. Dasari , W. Choi , Z. Yaqoob , P. So , Phys. Rev. Appl. 2014, 1, 014002. 25419536 10.1103/PhysRevApplied.1.014002 PMC4236915

[10] F. Charrière , A. Marian , F. Montfort , J. Kuehn , T. Colomb , E. Cuche , P. Marquet , C. Depeursinge , Opt. Lett. 2006, 31, 178. 16441022 10.1364/ol.31.000178

[11] A. Kuś , M. Dudek , B. Kemper , M. Kujawińska , A. Vollmer , J. Biomed. Opt. 2014, 19, 046009. 24723114 10.1117/1.JBO.19.4.046009

[12] F. Charrière , N. Pavillon , T. Colomb , C. Depeursinge , T. J. Heger , E. A. D. Mitchell , P. Marquet , B. Rappaz , Opt. Express 2006, 14, 7005. 19529071 10.1364/oe.14.007005

[13] M. Habaza , B. Gilboa , Y. Roichman , N. T. Shaked , Opt. Lett. 2015, 40, 1881. 25872098 10.1364/ol.40.001881

[14] K. Kim , J. Yoon , Y. K. Park , Optica 2015, 2, 343.

[15] P. Müller , M. Schürmann , C. J. Chan , J. Guck , Proc. SPIE 2015, 9548, 95480U.

[16] S. S. Kou , C. J. R. Sheppard , Opt. Lett. 2008, 33, 2362. 18923623 10.1364/ol.33.002362

[17] O. Haeberlé , K. Belkebir , H. Giovaninni , A. Sentenac , J. Mod. Opt. 2010, 57, 1362.

[18] V. Stanislas , J. Flügge , J. J. Delaunay , O. Haeberlé , Cent.Eur. J. Phys. 2011, 9, 969.

[19] J. Gimsa , P. Marszalek , U. Loewe , T. Y. Tsong , Biophys. J. 1991, 60, 749. 1835890 10.1016/S0006-3495(91)82109-9 PMC1260126

[20] C. Reichle , K. Sparbier , T. Müller , T. Schnelle , P. Walden , G. Fuhr , Electrophoresis 2001, 22, 272. 11288894 10.1002/1522-2683(200101)22:2<272::AID-ELPS272>3.0.CO;2-K

[21] M. Kirschbaum , C. R. Guernth‐Marschner , S. Cherré , A. de Pablo Peña , M. S. Jaeger , R. A. Kroczek , T. Schnelle , T. Mueller , C. Duschl , Lab Chip 2012, 12, 443. 22124613 10.1039/c1lc20818g

[22] R. Pethig , G. H. Markx , Trends Biotechnol. 1997, 15, 426. 9351287 10.1016/S0167-7799(97)01096-2

[23] T. Schnelle , T. Muller , G. Fuhr , Med. Biol. Eng. Comput. 1999, 37, 264. 10396833 10.1007/BF02513297

[24] M. Kirschbaum , M. S. Jaeger , T. Schenkel , T. Breinig , A. Meyerhans , C. Duschl , J. Chromatogr., A 2008, 1202, 83. 18619604 10.1016/j.chroma.2008.06.036

[25] P. R. C. Gascoyne , J. Noshari , T. J. Anderson , F. F. Becker , Electrophoresis 2009, 30, 1388. 19306266 10.1002/elps.200800373 PMC3754902

[26] I. Guido , C. Xiong , M. S. Jaeger , C. Duschl , Microelectron. Eng. 2012, 97, 379.

[27] J. Voldman , Annu. Rev. Biomed. Eng. 2006, 8, 425. 16834563 10.1146/annurev.bioeng.8.061505.095739

[28] N. T. Shaked , Opt. Lett. 2012, 37, 2016. 22660106 10.1364/OL.37.002016

[29] P. Girshovitz , N. T. Shaked , Opt. Express 2013, 21, 5701. 23482143 10.1364/OE.21.005701

[30] A. C. Kak , M. Slaney , Principles of Computerized Tomographic Imaging, IEEE Press, USA 1988.

[31] X. J. Liang , A. Q. Liu , C. S. Lim , T. C. Ayi , P. H. Yap , Sens. Actuators 2007, 133, 349.

[32] S. D. Douglas , F. Tuluc , in Williams Hematology, 6th ed. (Eds: Beutler E., Lichtman M. A., Coller B. S., Kipps T. J.), McGraw‐Hill, New York 2010, Ch. 59 and 67.

[33] P. Girshovitz , N. T. Shaked , Opt. Lett. 2014, 39, 2262. 24978968 10.1364/OL.39.002262

[34] P. Girshovitz , N. T. Shaked , Opt. Express 2015, 23, 8773. 25968715 10.1364/OE.23.008773

[35] G. Dardikman , M. Habaza , L. Waller , N. T. Shaked , Opt. Express 2016, 24, 11839. 27410107 10.1364/OE.24.011839

[36] M. Kirschbaum , M. S. Jaeger , C. Duschl , Lab Chip 2009, 9, 3517. 20024031 10.1039/b911865a

[37] R. S. Thomas , H. Morgan , N. G. Green , Lab Chip 2009, 9, 1534. 19458859 10.1039/b819267g

[38] M. S. Jaeger , T. Mueller , T. Schnelle , J. Phys. D: Appl. Phys. 2006, 40, 95.

[39] H. Kern , A. Klein‐Vehne , A. Pfennig , T. Müller , G. Kauselmann , B. Zevnik , G. Gradl , presented at 2nd Annual Meeting of the Int. Society for Stem Cell Research (ISSCR), Boston, USA 2004.

