# Supplementary material for: Rapid 3D Refractive‐Index Imaging of Live Cells in Suspension without Labeling Using Dielectrophoretic Cell Rotation
Source: Adv Sci (Weinh). 2016 Oct 21;4(2):1600205. doi: 10.1002/advs.201600205 (PMC5323858; doi:10.1002/advs.201600205)
Supplement: Supplementary file 1 — Supplementary [file ADVS-4-na-s001.pdf]

Copyright WILEY-VCH Verlag GmbH & Co. KGaA, 69469 Weinheim, Germany, 2016.

ADVANCED SCIENCE
Open Access

Supporting Information

for *Adv. Sci.,* DOI: 10.1002/advs.201600205

Rapid 3D Refractive-Index Imaging of Live Cells in Suspension without Labeling Using Dielectrophoretic Cell Rotation

*Mor Habaza, Michael Kirschbaum, Christian Guernth-Marschner, Gili Dardikman, Itay Barnea, Rafi Korenstein, Claus Duschl, and Natan T. Shaked**

Supporting Information

Supplementary Video 1. Microelectrode design and cell handling procedure in the microfluidic channel, including cell trapping and rotation.

Supplementary Video 2. The cell off-axis interferogram from multiple directions of view during cell rotation in the DEP field cage.

Supplementary Video 3. 3-D renderings of the reconstructed refractive index map of an MCF-7 cancer cell.

Supplementary Video 4. 3-D renderings of the reconstructed refractive index map of a T cell.

Supplementary Video 5. 3-D renderings of the reconstructed refractive index map of a Monocyte.

Supplementary Video 6. 3-D renderings of the reconstructed refractive index map of a Neutrophil.
